# The crippling financial toxicity of cancer in the United States

**DOI:** 10.1080/15384047.2019.1632132

**Published:** 2019-07-10

**Authors:** Loren Collado, Isaac Brownell

**Affiliations:** Dermatology Branch, NIAMS, NIH, Bethesda, MD, USA

**Keywords:** Cancer, financial toxicity, economics, cost, survivorship

## Abstract

The financial cost of cancer treatment in the United States is astronomically high and is expected to rise. The economic burden of cancer care increasingly falls on the patients. Patients thus experience “financial toxicity” of cancer care that can have catastrophic consequences on health and quality of life. Here we examine the results reported by Gilligan et al. in their study of financial toxicity in US cancer patients over 50 years old. This study provided corroborating and compelling data about the financial toxicity experienced by cancer patients. Many questions remain, however, about the consequences of financial toxicity and the full reality of cancer care economics in America.

*Breaking Bad* is a critically acclaimed American television show about Walter White, who is diagnosed with lung cancer. Even though Walter worked two jobs and had health insurance, he still could not afford his medical care. To pay for his cancer treatment and to protect his family from financial burden, Walter illegally synthesized and sold methamphetamine. Although this is a fictional story, it highlights the reality of the economic burden of cancer in America. The 2010 costs of cancer care in the United States were estimated to be 124.57 billion, and this number is expected to increase to at least 157 billion by 2020.^1^ Patented anticancer drugs are often priced at 6-12k a month, with some exceeding 24k a month.^2^ Moreover, the medical community expects the next generation of treatments to be even more expensive. Just this past decade alone, the cost of anticancer drugs more than doubled.^2^ In addition to the increasing cost of cancer management, the economic burden of cancer is increasingly falling on the patients themselves.^3–6^ Insured patients pay a median of 500 a month for cancer care, while over half of cancer patients are considered underinsured, or spend more than 10% of their income on cancer treatment.^4^ In one report, 10% of the patients with Medicare spent 60% of their income on cancer treatment.^7^ This is not surprising when considering that Medicare patients can pay hundreds of dollars a month out of pocket for their cancer treatments.^8^ Unfortunately, direct medical costs are expected to increase by 40% for patients by 2020,^7^ thus exacerbating the financial burden of medical care for cancer patients in the United States.

The term “financial toxicity” describes the financial burden experienced by cancer patients and its consequences.^4^ Patients who cannot afford their cancer care, even with insurance, often use their savings and borrow money to pay for treatment.^6,9^ Moreover, 40–85% of patients also need to take time off work or quit their jobs during cancer treatment, exacerbating their financial strife.^6,9,10^ To pay for their care, patients often alter their lifestyles by reducing spending and by selling possessions or property.^3,4,8^ Unfortunately, these lifestyle changes do not protect patients from incurring debt or declaring bankruptcy.^6^ Financial toxicity has been studied across multiple cohorts in the United States. In their recent article, Gilligan et al. report on financial toxicity in patients diagnosed with cancer.^9^

Gilligan et al. characterized financial toxicity in 9.5 million newly diagnosed cancer patients over the age of 50 between 2000 and 2012.^9^ They reported that over 42% of patients fully depleted their assets and over 30% incurred debt (consumer, mortgage or home equity) by the second-year of their diagnosis.^9^ The odds of net worth depletion were higher for patients with worsening cancer, patients over 75 years old, as well as for patients with public or no insurance.^9^ By analyzing longitudinal data from the Health and Retirement Study (HRS), a large nationally representative cohort, their results provide compelling evidence of cancer-related financial toxicity across America. Nonetheless, many questions remain about the financial toxicity experienced by cancer patients.

The study by Gilligan et al. only considered financial toxicity in patients 50 years of age or older, thus excluding younger populations from their study.^9^ However, other reports suggest that cancer survivors 18–64 years old are more likely to experience financial toxicity than cancer survivors over 64 years old.^8,10,11^ This is presumably because younger patients have fewer assets,^8^ and most do not qualify for Medicare, which protects the elderly if they lack private insurance.^6^ Treatment costs are also higher for younger patients,^11^ potentially because their treatment regimens tend to be more aggressive.^11^ For example, the average stage IV breast cancer costs for patients 18–64 years old were ~190k, whereas the costs were ~120k for patients 65 years old and over.^11^ Given that patients under 65 years old account for half of all cancer diagnoses in America,^11^ it is essential to understand their financial hardships.

Additionally, by relying on the HRS, Gilligan et al. were not able to comment on financial toxicity in disenfranchised groups, such as homeless or undocumented patients.^9^ Undocumented immigrants have the lowest rate of health insurance in America, thus increasing their risk of financial toxicity due to cancer. This is because they are not eligible for Affordable Care Act exchanges or Medicaid benefits. Undocumented immigrants can get health coverage through their employers, however most take low paying jobs that do not offer health insurance.^12^ Similarly, 60% of the homeless people in the US are uninsured.^13^ Most homeless people have poor access to preventative care, six to ten times worse than the general population. Accordingly, 73% of the homeless people have at least one unmet health-care need.^13^ Multiple reports, including the Gilligan et al. study, show that private insurance is protective against financial toxicity.^9,10,14^ Given that these groups typically also lack the assets to pay for their medical care, it is expected that they have increased susceptibility to devastating financial toxicity when compared to other cohorts.

In their study, Gilligan et al. did not stratify financial toxicity based on cancer type or stage.^9^ However, cancer-related costs are known to vary by these factors.^1,5,11,15^ For example, brain, pancreas, ovarian, esophagus, and stomach cancers were more expensive in the initial phases of cancer care when compared to melanoma, prostate, and breast cancer.^1^ Patients with expensive cancers can expect to pay more in direct medical care costs. For example, women with melanoma pay ~900 a year, whereas women with pancreatic cancer spend nearly 9k a year for their medical care.^5^ Moreover, cancer care costs vary depending on the stage.^1,5,11,15^ Costs are nine times more expensive for patients with advanced cancer when compared to patients with early stage cancer.^5^ For example, average one-year costs for stage I lung cancer ranged from 44-50k whereas stage IV lung cancer averages 71-97k.^11^ Moreover, cancer care costs are reported to be higher at the end of life phase when compared to initial treatment.^5^ Depending on cancer type and stage, costs range between 20-100k during the first year after diagnosis, but the average cost increases to over 60k at the end of life.^5^ This evidence suggests that terminally ill patients experience worse financial toxicity. Notably, Gilligan et al. did not assess the financial toxicity experienced during end of life care because they omitted the ~30% of the cancer patients who died during the study.^9^ Consequently, the study does not examine the significant financial toxicity associated with fatal cancers.

Gilligan et al. also did not assess the influence of treatment regimen on financial toxicity.^9^ Cancers requiring protracted treatments are associated with higher costs and worse financial toxicity.^1,5,8^ Consistent with this, Gilligan et al. found a 7% increase in odds of net worth depletion for patients requiring continued treatment.^9^ However, cancer care costs vary depending on the treatment regimen. Factors such as, number of hospitalizations, cost of travel to appointments, oral versus intravenous drugs, surgery, chemotherapy or radiation therapy influence the cost of cancer care.^6,8,9,15^ Cancer drug prices alone vary widely.^2,8^ Many drugs are priced at over 10k a month while a few treatments such as pralatrexate, which is used to treat T cell lymphoma, costs 120k per course.^2^ Given that the prices of cancer treatments are rising, and yet some newer drugs offer only a modest increase in patient survival,^2,16^ oncologists are now starting to assess cost effectiveness of cancer treatments. For example, Memorial Sloan Kettering Cancer Center in New York City wrote an editorial in the *New York Times* explaining that they would not stock aflibercept, a colorectal cancer treatment, because it was twice as expensive and yet equally as effective as alternative drugs. Following this article, the pharmaceutical company dropped the price of aflibercept by 50%.^2,8,17^ By including cost effectiveness in medical decision-making, oncologists have the power to lower treatment costs, protect patients from financial toxicity, and pressure pharmaceutical companies to constrain drug prices.^2^

The financial toxicity of cancer goes beyond economic expenditure because it affects the quality of life and health of cancer patients even years after their initial diagnosis.^3,8^ About a quarter of cancer survivors report psychological hardship related to financial toxicity.^10^ Analogously, Gilligan et al. found poorer self-reported health associated with significant financial toxicity,^9^ but they did not study psychological hardships in detail. Importantly, financial toxicity can be a barrier to getting necessary medical care.^8,14,18^ When patients are unable to afford the exorbitant prices for life-saving treatment, they may decline or delay care.^14^ Among patients enrolled in copayment assistance programs, 20% took less medication than prescribed, 19% filled half of their prescriptions, and 24% did not fill prescriptions. Over 7% of the patients in these programs delayed procedures, testing, chemotherapy or clinic visits.^4^ It is likely that the protracted financial toxicity of treating cancer results in delays and omissions of healthcare, possibly contributing to the association between financial toxicity and worse cancer outcomes. Financial hardships can last up to five years after patients enter remission,^10^ presumably because cancer survivors continue to suffer from higher out-of-pocket costs when compared to patients without a history of cancer.^5–7,14^ Financial toxicity, therefore, continues to be a barrier to necessary medical care.^18^ In fact, it is estimated that two million cancer survivors failed to receive at least one medical service (medical, dental or mental health care) between 2003 and 2006 in the United States.^18^ Further understanding of how economic access influences health-care use will be needed to accurately estimate the real-world efficacy and cost effectiveness of cancer treatments.

Oncologists should not ignore the growing evidence that financial toxicity has enduring and pervasive negative consequences affecting cancer patients and survivors. Fortunately, oncologists recognize that patients deserve competent, affordable care, and that it is their responsibility to provide said care.^16^ Multiple resources are available from organizations like the American Cancer Society and the American Society of Clinical Oncology with tips on how oncologists can engage their patients in developing cost effective treatment regimens that they can afford.^8,11,14^ By using these resources and encouraging price negotiations with pharmaceutical companies, oncologists can transform cancer care in the United States for the sake of their patients.
